# Cancer of the Uterus

**Published:** 1902-06-28

**Authors:** 


### HOSPITAL CLINICS AND MEDICAL PROGRESS.

### CANCER OF THE UTERUS.

AMONG the many forms which cancer takes on, cancer of the uterus has for a long time appeared the most hopeless; indeed it is not too much to say that up to a comparatively few years ago a case in which cancer of the uterus could be definitely diagnosed was definitely condemned to a fatal issue. This distressing condition of affairs was due principally to two things—first, lateness of diagnosis, second, the limitation imposed upon treatment by the imperfect surgery of those days. The latter condition has been much modified during recent years, so much so in fact that one may safely say that, given a sufficiently early diagnosis, a very large proportion of the cases might be cured. Even as things are, when the disease is recognised at an early stage a not inconsiderable number of cases are being cured by operation. Early diagnosis and thorough removal seem then to be the keys by which the problem of cancer of the uterus is to be solved. Writing on this subject Dr. Arthur Lewers[1] says that the difficulties that lie in the way of diagnosing cancer of the uterus in a stage so early as to be probably curable are many. First, is the fatal tradition clinging to the name of the disease. It has for so long been considered that a diagnosis of cancer of the uterus was equivalent to a sentence of death that it is not to be wondered at that many practitioners should hesitate to insist on making a proper examination and should be content to treat symptoms as they arise. For, after all, "what great harm could there be in postponing the certain diagnosis of a disease for which practically nothing could be done," even if discovered? Again, delay in the recognition of the disease may be the fault of the patient, for when suspicious local symptoms occur, such as bleeding between the periods or after the menopause, patients will not rarely let things drift in the hope that there may be nothing serious amiss. This unfortunate delay is also often due to the fact that in the lay mind pain is regarded as a necessary accompaniment of cancer, so that if the patient has no pain she is apt to comfort herself with the reflection that whatever else her ailment may be, at least it is not cancer. Yet, as is well known, in cases of cancer of the cervix, which constitute the large majority of those of cancer of the uterus pain comes on later, and at a time when the disease has passed beyond the reach of radical treatment. Lastly, as tending to retard early diagnosis, is the curious fact that many patients, for a considerable time after the commencement of cancer of the uterus, look quite well, having a good colour, and not only being well nourished, but in many instances being excessively fat. From the point of view of treatment, patients with cancer of the uterus may be divided into "early" and "advanced" cases. In regard to the former the question which presses for an answer is What is the best method for ensuring the most complete removal of the disease? while in regard to the latter one has to ask whether adventurous surgery, upon the same lines as have proved so successful in cancer of the breast, namely, by extensive removal of glands and lymphatic connections is possible, and, if possible, hopeful? In regard to early cases, Dr. Lewers says that, bearing in mind the direction in which cancer of the cervix usually spreads, it would seem to follow that a complete removal of the whole cervix at the level of the internal os, or even with a piece of the lower part of the body of the uterus as well, would do as much for the patient, and give her as good a chance of freedom from recurrence, as removal of the whole uterus. This view is borne out by the after-histories of cases in which this course has been adopted. At the same time it must not be forgotten that there are certain troublesome sequelæ which are apt to follow this operation, especially those which arise from contraction of the opening into the uterus, all of which are avoided by vaginal hysterectomy; and in view of the greatly reduced mortality which now attends the latter operation, Dr. Lewers recommends vaginal hysterectomy as the method to be adopted in all cases of cancer of the cervix which appear suitable for a radical operation. So much for early cases, which of course must be really early ones if they are to turn out well. The questions which arise in regard to the more advanced cases are much more serious and open to doubt. Dr. Lewers says that "a radical operation, that is to say, an operation undertaken with the hope of giving the patient either permanent immunity from the disease, or at all events a prolonged interval of freedom from the symptoms, should, in my opinion, be restricted to cases in which, so far as physical examination can ascertain, the malignant growth has not spread beyond the anatomical limits of the uterus." Many operators, however, have sought to extend the scope of surgery to cases in which the broad ligaments, or the vagina, or both are known to be affected, and Dr. Lewers has carefully analysed the published results of such attempts with the object of ascertaining whether they are such as to justify these more extensive operations. As the outcome of his investigations he concludes that, "at all events, when the broad ligaments are involved it is useless to operate. The patient will obtain neither permanent benefit nor any prolonged period of immunity from the disease, and the same is true when the vagina is at all extensively involved." Moreover, it must not be forgotten that it is evident, from the account given of the operations required in these cases, that in the majority of them the operation was of an exceedingly severe character, and that the patient ran an immediate risk very much greater than that incurred in a vaginal hysterectomy upon an early case. Much the same principle should be observed in deciding whether or not to do a radical operation in cases of cancer of the body of the uterus; that is to say the uterus should be removed in those cases only in which the disease seems to be limited to the uterus itself. In these cases the abdominal route seems to offer many advantages. Now for the after results. Dr. Lewers says that up to April, 1899, he had performed 33 supra-vaginal amputations of the cervix for cancer, and 28 vaginal hysterectomies for cancer of the cervix—61 operations for cancer of the cervix. Of these cases 8 treated by the supra-vaginal amputation and 6 treated by vaginal hysterectomy are known to have remained free from recurrence from 4 to 15 years later, and if we may regard an immunity of this duration as equivalent to a cure of the disease "14 out of 61 cases of cancer of the cervix may be considered cured, that is to say, 23 per cent." Again, Dr. Lewers says that in the cases of vaginal hysterectomy for cancer of the body of the uterus as detailed in his paper "the results are 5 out of 11 cases, or 45 per cent. remaining well from 4 to 7 years after the operation." The mortality in the series of 33 cases treated by supra-vaginal amputation was *nil*, that in the series of 40 vaginal hysterectomies was 3, or 7·5 per cent.; or, putting the two sets of cases together, the mortality of the 73 operations was 4 per cent. One need hardly say how encouraging all this is. On the other hand, the better the results obtained by early operation the greater the importance of early diagnosis. To this subject we shall return, for on it everything seems to hinge.

[1] Practitioner, June 1902.

